# Cohort Profile Update: The Intergenerational Childhood Adversity and Lifetime Morbidity (I-CALM) study, an extension of the Mater-University of Queensland Study of Pregnancy (MUSP)

**DOI:** 10.1093/ije/dyaf151

**Published:** 2025-09-01

**Authors:** Claudia Bull, Mike Trott, Jake Najman, Natasha Reid, Lakshmi Neelakantan, Rebecca Moran, Anne Edwards, Steve Kisely

**Affiliations:** Queensland Centre for Mental Health Research, Health, Medicine and Behavioural Sciences Faculty, The University of Queensland, QLD, Australia; Princess Alexandra Hospital Southside Clinical Unit, Greater Brisbane Clinical School, Medical School, Health, Medicine and Behavioural Sciences Faculty, The University of Queensland, QLD, Australia; Queensland Centre for Mental Health Research, Health, Medicine and Behavioural Sciences Faculty, The University of Queensland, QLD, Australia; Princess Alexandra Hospital Southside Clinical Unit, Greater Brisbane Clinical School, Medical School, Health, Medicine and Behavioural Sciences Faculty, The University of Queensland, QLD, Australia; Metro South Addiction and Mental Health Service, Metro South Health, Brisbane, QLD, Australia; School of Public Health, The University of Queensland, Brisbane, QLD, Australia; School of Social Sciences, The University of Queensland, Brisbane, QLD, Australia; Centre for Health Services Research, The University of Queensland, Brisbane, QLD, Australia; Child Health Research Centre, The University of Queensland, Brisbane, QLD, Australia; Population Mental Health Unit, Centre for Mental Health and Community Wellbeing, School of Population and Global Health, The University of Melbourne, Melbourne, VIC, Australia; Big Anxiety Research Centre, University of New South Wales, Sydney, NSW, Australia; Queensland Family and Child Commission, Queensland Government, Brisbane, QLD, Australia; Princess Alexandra Hospital Southside Clinical Unit, Greater Brisbane Clinical School, Medical School, Health, Medicine and Behavioural Sciences Faculty, The University of Queensland, QLD, Australia; Metro South Addiction and Mental Health Service, Metro South Health, Brisbane, QLD, Australia; Departments of Psychiatry, Community Health and Epidemiology, Dalhousie University, Canada

**Keywords:** birth cohort, longitudinal, administrative health data, data linkage, intergenerational trauma, child maltreatment, perinatal, emergency department, admission, mental health

Key Features

- Child maltreatment (CM) is a global public health crisis with severe, enduring consequences, but the intergenerational impacts remain poorly researched.
- The Mater-University of Queensland Study of Pregnancy (MUSP) began in 1981, collecting data on 6753 pregnant women (Generation 1) and their 7223 babies (Generation 2).
- In 2000, CM cases related to MUSP Generation 2 were linked to their records.
- The Intergenerational Childhood Adversity and Lifetime Morbidity (I-CALM) study aims to understand the associations between maternal experiences of CM, and health outcomes and service use in the next generation (Generation 3).
- I-CALM includes 1696 women (Generation 2) who gave birth to 3296 children (Generation 3) between 2008 and 2024.
- The average age of Generation 3 as of 30 June 2024 was 10.6 ± 4.1 years.
- I-CALM includes diverse health outcomes and service use measures from perinatal, inpatient, emergency, and community mental health datasets.
- Contact Dr Claudia Bull (claudia.bull@uq.edu.au) regarding opportunities for collaboration.

## The original cohort

The Mater-University of Queensland Study of Pregnancy (MUSP) study commenced as a 3- to 5-year longitudinal cohort study of 6753 pregnant women (Generation 1) birthing at the Mater Hospital in Brisbane, Australia, between 1981 and 1983 [1]. It also sought to follow up the 7223 babies (Generation 2) born to these women at 6 month postpartum [1]. MUSP has since evolved into one of Australia’s longest-running longitudinal birth cohort studies [2], with completed follow-up studies at 5, 14, 21, 27, 30, and 40 years [3].

In 2000, notified and substantiated child maltreatment (CM) cases reported to the Queensland Department of Families, Seniors, Disability Services and Child Safety were confidentially linked to Generation 2 records [4]. Notified instances of CM occur when contact has been made to an authorised department by people or other bodies alleging child abuse or neglect, child maltreatment, or harm to a child [5]. Substantiated instances of CM occurred when a notification was investigated and concluded that there was reasonable cause to believe the child had been, was being, or was likely to be abused, neglected, or otherwise harmed [5].

**Figure 1.**
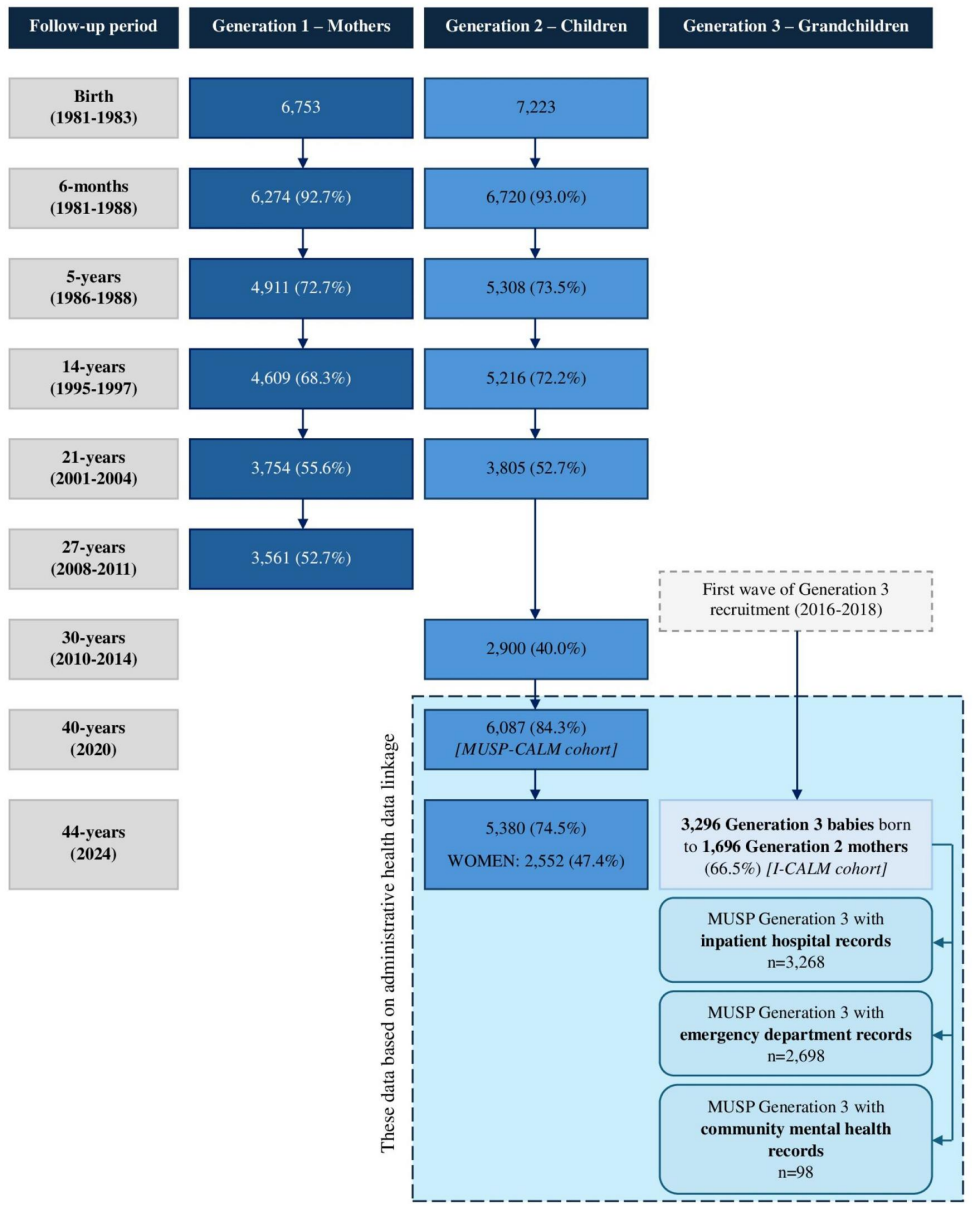
MUSP, MUSP-CALM, and I-CALM cohort composition at each phase of data collection.

In 2023, the MUSP-Childhood Adversity and Lifetime Morbidity (MUSP-CALM) study was conceived, which linked public and private hospital admissions data, emergency department (ED) presentations data, and community mental health data to Generation 2, alongside their CM records. By linking to administrative health data, the MUSP-CALM study was able to increase the MUSP Generation 2 cohort size from 2900 participants (40.0%) at the 30-year follow-up (based on self-reported data) to 6087 participants (84.3%), significantly reducing the attrition rates (Figure 1). The loss to follow-up at 30 years was attributed largely to the fact that Generation 2 had entered a busy phase of life (28–30 years old with work and childcare commitments) [3]. The requirement for early-morning fasting blood glucose collection at the 30-year follow-up study may also have deterred participants, as it posed a practical inconvenience [3]. Those lost to follow-up were more likely to be younger, less educated, unmarried, receiving welfare benefits, not own a house, smoke, and be born in a non-English-speaking country [6]. Thus, the MUSP-CALM linkage also allowed a comprehensive examination of the consequences of CM on health outcomes and health service use up to 40 years of age [7–14].

## What is the reason for the new data collection?

The Intergenerational Childhood Adversity and Lifetime Morbidity (I-CALM) study was designed to explore the intergenerational consequences of CM by confidentially linking the administrative health records of Generation 3 to women in the MUSP Generation 2 cohort. Recent Australian research indicates that nearly two-thirds of Australian adults self-report experiencing abuse or neglect before the age of 18 years [15]. Self-reported CM data from the MUSP cohort collected at 30-year follow-up by using the Childhood Trauma Questionnaire Short-Form (CTQ-SF) revealed that 24.5% self-reported experiencing any type of CM [16], with 17.9% reporting physical abuse, 24.5% reporting emotional abuse, 40.8% reporting neglect, and 11.1% reporting sexual abuse [11]. Notified and substantiated CM cases in the MUSP cohort indicate a more conservative estimate. Approximately 11% of children experience at least one notified CM incident and 7.1% experience at least one substantiated CM incident [9]. These estimates more closely align with data from the Australian Institute of Health and Welfare, which indicate that, in 2022–23, 1 in 32 Australian children came into contact with the child protection system (∼3.1%) and 121, 000 children (21 per 1000) were the subject of an investigation (2.1%) [17].

While the prevalence of CM remains a topic of debate [18], its financial burden in Australia is nonetheless considerable, exceeding $5 billion annually [19]. Moreover, the lifetime health and social consequences are substantial [14]. The MUSP and MUSP-CALM studies have reported that individuals who experienced one or more forms of CM were significantly more likely to experience negative long-term educational and employment outcomes in young adulthood [20, 21]; poorer psychological and mental health outcomes, including suicidal ideation and behaviour, anxiety, depression, post-traumatic stress disorder (PTSD), psychosis, delinquency, attention-deficit/hyperactivity disorder, and aggressive behaviour [22–27]; greater susceptibility to intimate partner violence and harassment [22–25]; alcohol and other substance-use disorders and addiction [7, 8, 28–30]; young pregnancy [31]; pregnancy miscarriage [31]; greater risk of obesity [32]; and poorer sleep quality [33].

There is a body of evidence to support the theory of intergenerational CM transmission [34–36]. That being, maltreatment begets maltreatment, whether that is in a homotypic pattern of transmission (i.e. an emotionally abused woman is more likely to have a child who experiences emotional abuse) or a heterotypic pattern of transmission (i.e. an emotionally abused woman is more likely to have a child who experiences physical abuse) [37]. Yet there remains a critical knowledge gap regarding the intergenerational consequences of CM in terms of health outcomes and patterns of health service use. As such, I-CALM employed a similar data linkage methodology as MUSP-CALM to anonymously identify members of the Generation 3 cohort. Based on the number of Generation 2 cohort members who had linkable administrative health data in 2024, we identified women who had given birth in Queensland and subsequently identified the babies born to these women (Generation 3).

## What will be the new areas of research?

The following research question will be addressed through the I-CALM study: Are maternal experiences of CM associated with health outcomes and health service use in the next generation? We hypothesise that maternal experiences of CM will be strongly associated with poorer health outcomes in Generation 3, as well as increased health service use. We also hypothesise that maternal mental illness will be strongly associated with poorer health outcomes in Generation 3, as well as increased health service use.

This study was registered as a retrospective observational study with the Australian New Zealand Clinical Trials Registry (registration number: ACTRN12624001423505). Human Research Ethics Committee (HREC) approval was obtained from the Metro South HREC (reference number: HREC/2024/QMS/107240) and the University of Queensland HREC (project number: 2024/HE002338).

## Who is in the cohort?

**Figure 2.**
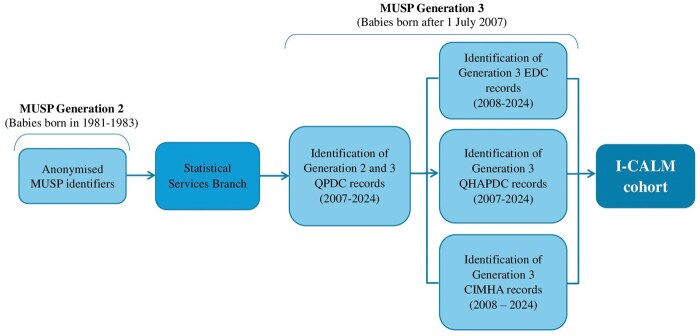
Flow diagram of the I-CALM data linkage between the MUSP, the QPDC, the Queensland Hospital Admitted Patient Data Collection (QHAPDC), the Emergency Data Collection (EDC), and the Consumer Integrated Mental Health and Addiction (CIMHA) datasets.

To identify the I-CALM Generation 2 and 3 cohorts, MUSP Generation 2 identifiers (limited to name and date of birth) were supplied by the MUSP data custodian to the Queensland Statistical Services Branch (SSB, Figure 2). The SSB subsequently identified and matched relevant Generation 3 records via the Queensland Perinatal Data Collection (QPDC) by using a Master Linkage File (MLF). Matching records by using an MLF is highly accurate and involves deterministic matching as opposed to probabilistic matching (i.e. a review of all possible matches is undertaken rather than using matching thresholds) [38]. A quality assurance process involving a manual review of any ‘grey areas’ identified was also undertaken by an independent member of the data linkage team [38]. A data linkage report was subsequently provided to the research team describing the linkage methodology employed, the constituents of Generations 2 and 3, and the linked Generation 3 data.

Figure 1 illustrates how the I-CALM Generation 2 and 3 cohorts were derived and how they relate to the MUSP and MUSP-CALM studies. Of the 5380 members (74.5%) of the Generation 2 cohort who had linkable data in 2024, 2552 (47.4%) were women and 1696 (66.5%) gave birth in the state of Queensland, Australia between 1 January 2008 and 30 June 2024. Table 1 shows that most women gave birth to their first child between the ages of 25 and 29 years (56.4%). There were 3296 babies born, representing Generation 3 of the I-CALM cohort. At the latest data extraction (30 June 2024), the mean age of the Generation 3 cohort was 10.6 years (SD = 4.1).

**Table 1.** Description of the I-CALM Generation 2 and 3 cohort sizes and ages.

Cohort characteristic	Generation 2	Generation 3
Cohort size [*n*, (%)]	1696 (100)	3296 (100)
**Sex [*n*, (%)]**		
Women	1696 (100)	1628 (49.4)
Men		1668 (50.6)
**Age at first birth (years) [*n*, (%)]**		
20–24	106 (6.3)	
25–29	956 (56.4)	
30–34	458 (27.0)	
35–39	151 (8.9)	
40–44	25 (1.5)	

## What has been measured?

The data linkage for I-CALM provides new health outcomes and health service use measures for Generation 3 based on perinatal, admitted hospital, ED, and community mental health service data collections. It also provides new health outcomes and health service use measures for Generation 2 based on perinatal data collection. Sociodemographic measures for both Generations 2 and 3 are also provided. Table 2 describes these new measures.

**Table 2.** New health outcomes and service use measures enabled through data linkage for the I-CALM study.

Data source	New health outcome measures	New health service use measures	Socio-demographic measures
QPDC	Generation 2: Previous number of pregnancies (including number in which the outcome was a live birth, stillbirth^a^, or abortion/miscarriage/ectopic pregnancy/hydatidiform mole) Parity (total number of live births) Presence of pre-existing medical conditions and other conditions arising during the current pregnancy (based on ICD-10-AM codes) Pregnancy-related complications arising in the period immediately preceding delivery (based on ICD-10-AM codes) Postnatal depression as captured by the EPDS Damage to the perineum (if any) and the extent, e.g. degree of laceration (based on ICD-10-AM codes) Generation 3: The manner in which labour started, e.g. spontaneous, induced, or nil due to caesarean section Presentation at birth, e.g. cephalic, vertex, or breech Complications during labour and delivery (based on ICD-10-AM codes) Gestational age at birth (in weeks) Weight at birth Plurality, e.g. singleton, twins APGAR score 1 and 5 minutes after birth Resuscitation required Presence of neonatal morbidity identified at time of birth up to discharge from birth event (or 28 days) (based on ICD-10-AM codes) Presence of congenital anomalies (anatomical defects or chromosomal abnormalities) identified at time of birth up to discharge from birth event (based on ICD-10-AM codes)	Generation 2: Number of birth-related hospital stays Length of stay per birth-related hospital stay Type of antenatal care received during pregnancy, e.g. private vs public, midwifery-led vs obstetric-led) Number of previous caesarean sections Receipt of smoking-cessation advice Total number of antenatal-care visits for the current pregnancy Whether the current pregnancy was the result of ART and what method was used Procedures or operations performed on the woman during pregnancy, labour, delivery, or puerperium Antenatal screening for domestic and family violence Antenatal screening for illicit drug use Generation 3: Length of stay for the birth event Way in which the baby was delivered, e.g. vaginal non-instrumental, caesarean section, vacuum, forceps) Admission to SCN or NICU	Generation 2: Age (in 5-year groups) First Nations status at time of birth Marital status at time of birth Smoking status during pregnancy (measured as ‘at any time’, ‘before 20 weeks’ gestation’, and ‘after 20 weeks’ gestation’) Alcohol-consumption status of Generation 2 during pregnancy (measured as ‘at any time’, ‘before 20 weeks’ gestation’, and ‘after 20 weeks’ gestation’) Total number of standard drinks consumed on a typical day (measured as ‘at any time’, ‘before 20 weeks’ gestation’, and ‘after 20 weeks’ gestation’) Frequency of alcohol consumption (measured as ‘at any time’, ‘before 20 weeks’ gestation’, and ‘after 20 weeks’ gestation’) BMI at first antenatal appointment ARIA (measure of residential geographical remoteness) SEIFA (index of relative socio-economic advantage and disadvantage) Generation 3: Sex First Nations status
QHAPDC	Generation 3 only (based on ICD-10-AM codes): Severe mental illnesses Schizophrenia, schizoaffective, and other psychotic disorders (F20, F22, F23, F24, F25, F25.0, F25.1, F25.2, F25.8, F25.9, F28, F29) Severe or psychotic affective disorders (F30, F31, F32.2, F32.3) Psychotic disorders related to substance use (F10.5, F11.5, F12.5, F13.5, F14.5, F15.5, F15.50, F15.51, F15.59, F15.70, F16.5, F17.5, F18.5, F19.5, F19.7) Common mental disorders Depressive and other mood disorders (F32.0, F32.1, F32.8, F32.9, F33.0, F33.1, F33.4, F33.8, F33.9, F34, F38, F39) Phobic anxiety disorders (F40, F40.1, F40.2, F40.8, F40.9) Reaction to severe stress, e.g. acute stress reaction, PTSD (F43.0, F43.1, F43.8, F43.9) Adjustment disorders (F43.2) Other anxiety disorders, e.g. obsessive–compulsive, dissociative, and somatoform disorders (F41, F42, F44, F45, F48) Personality disorders Cluster A (F21, F60.0, F60.1) Cluster B (F60.2, F60.3, F60.30, F60.31, F60.4) Cluster C (F60.5, F60.6, F60.7) Other personality disorders (F60.8, F60.09, F60.9, F61, F62, F62.0, F62.1, F62.8, F62.9, F68.0, F68.1, F68.8, F69) Alcohol-use disorders Mental and behavioural disorders due to use of alcohol (F10) Counselling and surveillance for alcohol-use disorder (Z71.4) Substance-use disorders Mental and behavioural disorders due to use of opioids (F11) Mental and behavioural disorders due to use of cannabinoids (F12) Mental and behavioural disorders due to use of sedatives or hypnotics (F13) Mental and behavioural disorders due to use of cocaine (F14) Mental and behavioural disorders due to use of other stimulants, including caffeine (F15) Mental and behavioural disorders due to use of hallucinogens (F16) Mental and behavioural disorders due to use of tobacco (F17) Mental and behavioural disorders due to use of volatile solvents (F18) Mental and behavioural disorders due to multiple drug use and use of other psychoactive substances (F19) Harmful use of non-dependence-producing substances: antidepressants; laxatives; analgesics; antacids; vitamins; steroids or hormones; specific herbal or folk remedies; other substances that do not produce dependence; unspecified harmful use of non-dependence producing substance (F55) Counselling and surveillance for drug-use disorder (Z71.5) Counselling for tobacco-use disorder (Z71.6) Other childhood-onset disorders Mental retardation (F70, F71, F72, F73, F78, F79) Disorders of psychological development, e.g. disorders of speech and language, pervasive developmental disorders (F80, F81, F82, F83, F84, F88, F89) Childhood behavioural, e.g. conduct and hyperkinetic disorders, mixed disorders of conduct and emotion (F90, F91, F92) Other childhood-onset disorders, e.g. emotional disorders, disorders of social functioning, tic disorders (F93, F94, F95, F98) Deliberate self-harm and suicidal ideation (R45.81, T39.1, T42.4, T43.9, T43.5, X60–X84)	Generation 3 only: Number of hospital admissions Length of stay per admission Admission to ICU Length of stay in ICU Length of stay as an admitted patient/resident within a designated psychiatric unit	Generation 3 only: ARIA SEIFA
EDC	As listed above	Generation 3 only: Number of ED presentations Length of stay per ED presentation Triage category Mode of arrival at ED	Generation 3 only: ARIA SEIFA
CIMHA	Nil	Generation 3 only: Number of community MH appointments	Nil

a A stillbirth is classified as a birth that did not progress beyond 20 weeks’ gestation and was <400 grams; APGAR = Appearance, Pulse, Grimace, Activity and Respiration; ARIA = Accessibility/Remoteness Index of Australia; ART = artificial reproductive technology; BMI = body mass index; CIMHA = Community Integrated Mental Health Application; EDC = Emergency Data Collection; EPDS = Edinburgh Postnatal Depression Scale; ICD-10-AM = International Statistical Classification of Diseases and Related Health Problems, Tenth Revision, Australian Modification; ICU = intensive care unit; MH = mental health; NICU = neonatal intensive care unit; QHAPDC = Queensland Hospital Admitted Patient Data Collection; SCN = special care nursery; SEIFA = Socio-Economic Indexes for Areas.

## What has it found? Key findings and publications

The I-CALM data linkage was completed in January 2025. Table 3 provides preliminary descriptive statistics. Nearly all Generation 3 cohort members were admitted to hospital at least once between 1 January 2008 and 30 June 2024. Over three-quarters presented to an ED at least once and 2.0% of the Generation 3 cohort ever accessed community mental health services.

**Table 3.** Preliminary descriptive statistics for Generation 3 health service use and Generation 2 experiences of CM.

Generation 3—Health service use	
**Hospitalisations**	
Any inpatient admissions [*n*, (%)]	3268 (99.2)^a^
Average number of inpatient admissions (mean ± SD)	2.8 ± 4.4
Average hospital length of stay in days (mean ± SD)	2.6 ± 2.4
**ED presentations**	
Any ED presentations [*n*, (%)]	2698 (81.9)
Average number of ED presentations (mean ± SD)	4.1 ± 6.8
Average ED length of stay in hours (mean ± SD)	2.2 ± 1.6
**Community MH appointments**	
Any community MH appointments [*n*, (%)]	67 (2.0)
Average number of community MH appointments (mean ± SD)	0.03 ± 0.3

Generation 2—Experiences of CM	
**Allegations of abuse and/or neglect**	
Any type of allegation [*n*, (%)]	175 (10.3)
Emotional abuse allegations [*n*, (%)]	94 (5.5)
Neglect allegations [*n*, (%)]	98 (5.8)
Physical abuse allegations [*n*, (%)]	98 (5.8)
Sexual abuse allegations [*n*, (%)]	81 (4.8)
Age of first allegation (mean ± SD)	9.4 ± 4.6
**Substantiated allegations of abuse and/or neglect**	
Any type of substantiated allegation [*n*, (%)]	106 (6.3)
Substantiated emotional abuse [*n*, (%)]	56 (3.3)
Substantiated neglect [*n*, (%)]	48 (2.8)
Substantiated physical abuse [*n*, (%)]	55 (3.2)
Substantiated sexual abuse [*n*, (%)]	46 (2.7)
Age of first substantiated allegation (mean ± SD)	8.0 ± 4.5
**Self-reported moderate-severe abuse and/or neglect [*n*, (%)]**	
Any type of self-reported CM	231 (13.6)
Self-reported emotional abuse	102 (6.0)
Self-reported emotional neglect)	107 (6.3)
Self-reported physical neglect	81 (4.8)
Self-reported physical abuse	76 (4.5)
Self-reported sexual abuse	113 (6.7)

a *n* = 28 records have missing data (all Generation 3 cohort members should have at least one admission record for the birth episode); CM = Child Maltreatment; SD = Standard Deviation; ED = Emergency Department; MH = Mental Health.

Maternal experiences of CM will be critical to addressing our research question. Approximately 10% of women in the Generation 2 cohort had any record of notified CM during childhood. Neglect and physical abuse were the most common subtypes. Over 6% of the Generation 2 cohort had any record of substantiated CM during childhood. Substantiated emotional abuse and physical abuse were the most common subtypes. Over 13% of the Generation 2 cohort self-reported any form of moderate to severe CM by using the CTQ-SF. Self-reported sexual abuse, emotional neglect, and emotional abuse were the most common subtypes.

## What are the main strengths and weaknesses?

The primary strength of the I-CALM study lies in the linkage between the MUSP Generation 2 records and administrative health data to identify Generation 3 cohort members. This linkage has supported the creation of a robust dataset with nuanced health outcomes and health service use details. Notably, the health outcomes and health service use data provide objective endpoints of a diverse nature with which we can address our overarching research question. Moreover, both agency-reported (notified and substantiated) and self-reported sources of CM data are available for Generation 2. This enables us to provide a more comprehensive understanding of intergenerational CM transmission consequences, which addresses calls to present multiple sources of CM data when available to better support public health surveillance [18].

A weakness of I-CALM is that Generation 2 solely comprises women who gave birth in Queensland between 1 January 2008 and 30 June 2024, necessarily enabling the identification of the Generation 3 cohort. There were 5380 Generation 2 cohort members at the 44-year follow-up (2024) who had linkable administrative health data and 52.6% were men (Figure 1). Resultantly, male members of Generation 2 who may have had children were unable to be identified, as only women’s details are recorded in the QPDC. Moreover, if Generation 2 women gave birth outside of Queensland, then they would not have been identified, as the QPDC is a state-based administrative dataset. There are also no partner data for women in Generation 2, which is limiting because partners are likely to have contributed to the health and wellbeing of Generation 3 members.

Finally, another weakness of I-CALM is the variability in available data across the generations. While rich sociodemographic and health service use data are available for Generations 2 and 3 through administrative health data linkage, no such data are available for Generation 1. This limits our ability to examine intergenerational influences beyond two generations and may constrain interpretations of broader familial or social patterns.

## Can I get hold of the data? Where can I find out more?

Collaboration with other researchers is welcomed. This may take any number of forms, including the preparation of research papers on specific topics, development of grant applications to support a specific research endeavour, and visits by colleagues with complementary interests. This will involve working with one or more of the I-CALM investigators.

The I-CALM data are not openly accessible. Some of the data are owned by the original MUSP investigators and the linked administrative health data are owned by Queensland Health. Access to these data is only permissible for ‘designated persons’ listed on an approved Public Health Act application; only C.B., M.T., J.N., and S.K. have permission to access the data. Collaborators who wish to access the data will be required to fund the necessary ethics and Public Health Act application changes. Please contact Dr Claudia Bull via e-mail (claudia.bull@uq.edu.au) if you would like more information about accessing the I-CALM data.

Details of the original MUSP investigations, including each follow-up and data collections, can be found at https://social-science.uq.edu.au/mater-university-queensland-study-pregnancy?p=0#0. Collaborators interested in these data are encouraged to contact Emeritus Professor Jake Najman via e-mail (j.najman@uq.edu.au).

## Ethics approval

Ethical approval for this study was obtained from the Metro South Research Ethics Committee (reference number: HREC/2024/QMS/107240) and the University of Queensland Human Research Ethics Committee (project number: 2024/HE002338). This research was conducted in accordance with the ethical principles outlined in the Declaration of Helsinki.

## Data Availability

Due to privacy, ethical, and legal considerations, the administrative health data cannot be shared without direct approval from relevant data custodians and the Office of Research and Innovation of Queensland Health. Contact details for Queensland Health custodians can be found at https://www.health.qld.gov.au/__data/assets/pdf_file/0034/843199/data_custodian_list.pdf. MUSP data are available from a third party on reasonable request. Contact details can be found at https://social-science.uq.edu.au/mater-university-queensland-study-pregnancy?p=5#5.
